# Coagulation profile and platelet parameters in pregnancy induced hypertension cases and normotensive pregnancies: A cross-sectional study

**DOI:** 10.1016/j.amsu.2022.104124

**Published:** 2022-07-10

**Authors:** Namita Bhutani, Varuna Jethani, Sumit Jethani, Karuna Ratwani

**Affiliations:** a Deptt. of Pathology, North DMC Medical College and Hindu Rao Hospital, Delhi, India; b Deptt. of Pulmonary Medicine, Himalayan Institute of Medical Sciences, Dehradun, Uttarakhand, India; c Deptt. of Community Medicine, North DMC Medical College and Hindu Rao Hospital, Delhi, India; d Deptt. of Obstetrics and Gynaecology, Apollo Hospital, New Delhi, India

## Abstract

• PIH is a leading cause of maternal and perinatal morbidity and mortality worldwide.
• Hypercoagulability is constantly associated with hypertensive disorders of pregnancy and particularly associated with pre-eclampsia.
• The aims and objectives of this study were to compare the platelet parameters and coagulation profile in normotensive pregnant females along with gestational hypertension cases and pre-eclamptia patients.

## 1 Introduction

Pregnancy induced hypertension (PIH) is defined as newly diagnosed hypertension which occurs in pregnancy after 20 weeks of gestation and it disappear following delivery of the baby. PIH leads to intrauterine growth restriction (IUGR) and fetal distress which can ultimately result in fetal death. Worldwide PIH is considered as a leading cause of maternal and perinatal morbidity and mortality and is an important health issue that needs to be dealt especially in developing countries. In India, prevalence of hypertensive disorders of pregnancy was reported in 7.8% cases whereas 5.4% of the study population had associated preeclampsia. HELLP (hemolysis, elevated liver enzymes and low platelet count) syndrome and eclampsia are the serious complications. Hypercoagulability is constantly associated with hypertensive disorders of pregnancy and particularly associated with pre-eclampsia [1].

Out of all the hematological changes that occur in PIH, thrombocytopenia is the most widely recognized hematological abnormality and the degree of thrombocytopenia increases with increasing severity of the disease [2]. Coagulation and fibrinolytic system changes which occurs during normal pregnancy cause a hypercoagulable state. There is possibility of increase in the hypercoagulable state of pregnancy in PIH too. Coagulation abnormalities associated with PIH increases the risk of bleeding complications, especially during operative delivery or during the placement of an epidural catheter for regional anesthesia [3]. Therefore coagulation profile tests with complete blood cell counts including platelet counts and platelet indices are essential in patients with a hypertensive disorder of pregnancy to look for evidence of Disseminated Intravascular Coagulation (DIC) and HELLP Syndrome [4].

Prothrombin time (PT) and APTT are most commonly performed tests in coagulation profile for the detection of coagulation defects. Both PT and APTT are considered as functional tests as they are used to measure enzymatic activities that lead to clot formation*.* The haemostatic system during pregnancy changes to a more procoagulant state and with lower levels of natural anticoagulants like protein C and S. An increase in levels of coagulation factor V, VII, VIII, IX, XII, fibrinogen and D-dimer are also seen. Gradually post-partum, haemostatic changes revert to a normal state [5]. This study was undertaken to assess the severity of coagulation changes in PIH cases by a method that is rapid and economical, so that these investigations will guide in the management of cases before the patients goes into life threatening complications. The aims and objectives of this study were to compare the platelet parameters and coagulation profile in normotensive pregnant females along with gestational hypertension cases and pre-eclamptia patients and also to detect platelet defects and coagulation failure early and manage its complications before it worsens.

## 2 Material and methods

The present cross-sectional study was conducted in Departments of Pathology and Obstetrics and Gynecology, in North Delhi Medical College and Hindu Rao Hospital, Delhi, over a period of two years (2019–21). A total of 104 patients were included in the study after obtaining the Ethical clearance from the Institutional Review Board, vide no. Dean/North DMC/MC/2021/1031 Dated 27-12-2021(Certificate attached).

The study included normotensive pregnant females, pregnant women with diagnosed pregnancy induced hypertension and pre-eclampsia cases with more than 20 weeks of gestation presenting in our hospital. Platelet parameters and Coagulation Profile were studied in total 52 PIH and Pre-eclampsia cases. Study groups were divided into following four groups comprising of normotensive pregnant females, females with Gestational hypertension/PIH, mild preeclampsia cases and severe preeclampsia cases. The control group included 52 normotensive pregnant women and rest 52 cases of the study group comprised of three groups as gestational hypertension, mild preeclampsia and severe preeclampsia respectively. Any pregnant women with known history of bleeding disorders, those on anticoagulant therapy, with abruptio placentae, IUD, established DIC, Pre Hypertensive cases and those in labour were excluded from the study.

For all the study groups’ coagulation profile and platelet parameters like Bleeding Time (BT), Clotting Time (CT), Prothrombin Time ((PT), Activated Partial Thrombin Time (APTT), Platelet Count, Mean Platelet Volume (MPV) and Platelet Distribution Width (PDW) were done. Relevant data were obtained from case files and compiled by a common proforma that included socio-demographic characteristics of patients, their obstetric history, signs and symptoms at the time of presentation, laboratory data, and maternal and perinatal outcomes. The data collection was followed by analysis of the collected data. Statistical analysis was done which were compared with various available previous studies. P < 0.05 was considered as significant statistical difference. Comparison was made using Chi square test. The statistical analyses were performed using SPSS version 22 for windows. The work has been reported in line with the STROCSS criteria [6].

## 3 Results

The distribution of the study group according to gravida showed that 23.8% of the patients with gestational hypertension, 17.31% of the patients with mild preeclampsia and 5.77% cases with severe eclampsia were primigravida, whereas 17.31% patients with gestational hypertension, 11.54% patients with mild preeclampsia and 5.77% of cases with severe pre-eclampsia were gravida 2. There were 7.69% cases of gestational hypertension, 3.85% of mild preeclampsia cases were gravida 3. A total of 5.77% of gestational hypertension and 7.69% cases of mild preeclampsia cases was multigravida. No case from severe preeclampsia was of multigravida status. There was no statistically significant difference between the gravida status of the patients with gestational hypertension, preeclampsia and eclampsia (Table 1).

**Table 1:** Shows distribution of cases according to Gravida.

Gravida	Gestational Hypertension n (%)	Mild Preeclampsia n (%)	Severe Preeclampsia n (%)
**Primigravida**	12(23.08%)	9(17.31%)	3(5.77%)
**Gravida 2**	9(17.31%)	6(11.54%)	3(5.77%)
**Gravida 3**	4(7.69%)	2(3.85%)	0(0.00%)
**Multigravida**	3(5.77%)	4(7.69%)	0(0.00%)
**Total**	28(53.85%)	18(34.62%)	6(11.54%)

The Present study showed the mean platelet count for normal pregnancy; gestational hypertension, mild preeclampsia and severe preeclampsia were 2.85 ± 0.73 lakhs/cumm, 2.01 ± 0.54 lakhs/cumm, 1.81 ± 0.46 lakhs/cumm, 1.66 ± 0 lakhs/cumm respectively. There was a statistically significant difference between the platelet count of patients with gestational hypertension, mild preeclampsia and severe preeclampsia (Table 2).

**Table 2:** Shows distribution of platelet count in study groups and control group.

Platelet count	Normal Pregnancy n (%)	Gestational hypertension n (%)	Mild Preeclampsia n (%)	Severe Preeclampsia n(%)
**less than 1 lac**	0(0.00%)	1(3.57%)	0(0.00%)	1(16.67%)
**1**–**2.5 lakhs**	3(5.77%)	23(82.14%)	15(83.33%)	5(83.33%)
**> 2.5 lakhs**	49(94.23%)	4(14.29%)	3(16.67%)	0(0.00%)
**Total**	52(100%)	28(100%)	18(100%)	6(100%)
**Mean ± SD**	**2.85 ± 0.73**	**2.01 ± 0.54**	**1.81 ± 0.46**	**1.66 ± 0.85**
**chi square**	**75.05**	**df=6**	**p-value<0.0001(S)**	

However, the mean platelet volume for normal pregnancy, gestational hypertension, mild preeclampsia and severe preeclampsia were 8.47 ± 1.93, 8.65 ± 0.97, 8.79 ± 0.68, 10.33 ± 1.07 fl respectively. Although mean platelet volume was found to be increased in 28.5% cases of gestational hypertension, 44.4% of the mild preeclampsia patients and in 50% of severe preeclampsia patients but this difference in mean platelet volume was not found to be statistically significant, as the p value was found out to be 0.07 (Table 3).

**Table 3:** Shows distribution of the study groups along with control group according to Mean platelet volume.

Mean Platelet Volume	Normal Pregnancy n (%)	Gestational hypertension n (%)	Mild Preeclampsia n (%)	Severe Preeclampsia n (%)
**Normal(8–10)**	50(96.15%)	20(71.43%)	10(55.56%)	3(50%)
**Increased(10–12)**	2(3.85%)	8(28.57%)	8(44.44%)	3(50%)
**Total**	52(100%)	28(100%)	18(100%)	6(100%)
**Mean ± SD**	**8.47 ± 1.93**	**8.65 ± 0.97**	**8.79 ± 0.68**	**10.33 ± 1.07**
**chi square**	**6.92**	**df=3**	**p-value=0.07(NS)**	

Although, the mean platelet distribution width for normal pregnancy, gestational hypertension, mild preeclampsia and severe preeclampsia were 10.90 ± 1.32, 11.33 ± 1.11, 11.96 ± 0.77, 11.7 ± 0.14 fl respectively. There was a no statistically significant difference, as the p value was found to be < 0.09 (Table 4).

**Table 4:** Shows distribution of the study groups along with control group according to Platelet distribution width.

Platelet distribution Width	Normal Cases n (%)	Gestational hypertension n (%)	Mild Preeclampsia n (%)	Severe Preeclampsia n (%)
**9–12**	45(86.54%)	18(64.29%)	12(66.67%)	4(66.67%)
**12–14**	7(13.46%)	10(35.71%)	06(33.33%)	2(33.33%)
**Total**	52(100%)	28(100%)	18(100%)	6(100%)
**Mean ± SD**	**10.90 ± 1.32**	**11.33 ± 1.11**	**11.96 ± 0.77**	**11.7 ± 0.14**
**Chi square**	**6.41**	**df=3**	**p-value=0.09(NS)**	

The mean prothrombin time of patients with gestational hypertension was 16.6 s, mild preeclampsia patients was 17.6 s and severe preeclampsia patients was 18.8 s 57.1% of cases with gestational hypertension, 83.3% of cases with preeclampsia and 100% cases of eclampsia showed prolonged prothrombin time. There was a statistically significant difference between the prothrombin time of patients with gestational hypertension, mild preeclampsia and severe preeclampsia (Table 5).

**Table 5:** Shows distribution of the study groups along with control group according to prothrombin time.

Prothrombin time	Normal Case n (%)	Gestational hypertension n (%)	Mild Preeclampsia n (%)	Severe Preeclampsia n (%)
**Normal(11–16)**	52(100.00%)	12(42.86%)	3(16.67%)	0(0.00%)
**Prolonged(>16)**	0 (0.00%)	16(57.14%)	15(83.33%)	6(100%)
**Total**	52(100%)	28(100%)	18(100%)	6(100%)
**Mean ± SD**	**12.95 ± 1.48**	**16.59 ± 1.44**	**17.61 ± 2.88**	**18.88 ± 0.0**
**Chi square**	**63.17**	**df=3**	**p value < 0.0001(S)**	

In present study, the mean APTT for normal pregnancy, gestational hypertension, mild preeclampsia and severe preeclampsia were 25.76 ± 2.99, 33.55 ± 2.44, 38.79 ± 2.52, 41.85 ± 0.00 s respectively. There was a statistically significant correlation between the APTT of patients with gestational hypertension, mild preeclampsia and severe preeclampsia as the p value was found to be < 0.0001 (Table 6).

**Table 6:** Shows distribution of the study groups along with control group according to APTT.

aPTT	Normal Pregnancy n (%)	Gestational hypertension n (%)	Mild Preeclampsia n (%)	Severe Preeclampsia n (%)
**Normal (25–37)**	52(100.00%)	14(50.00%)	6(33.33%)	0(0.00%)
**Prolonged (>37)**	0 (0.00%)	14(50.00%)	12(66.67%)	6(100%)
**Total**	52(100%)	28(100%)	18(100%)	6(100%)
**Mean ± SD**	**25.76 ± 2.99**	**33.55 ± 2.44**	**38.79 ± 2.52**	**41.85 ± 0.00**
**Chi square**	**52.36**	**df=3**	**p-** **value < 0.0001(S)**	

The mean bleeding time for normal pregnancy, gestational hypertension, mild preeclampsia and severe preeclampsia were 2.67 ± 0.86, 2.57 ± 0.68, 5.37 ± 0.72, 2.75 ± 1.06 s respectively. There was no statistically significant difference in the bleeding time between the patients with gestational hypertension, mild preeclampsia and severe eclampsia as the p-value was not significant (Table 7).

**Table 7:** Shows distribution of the study groups along with control group according to bleeding time.

Bleeding time	Normal Pregnancy n (%)	Gestational hypertension n (%)	Mild Preeclampsia n (%)	Severe Preeclampsia n (%)
**< 5 min**	38(73.08%)	20(71.43%)	7(38.89%)	3(50%)
**>5 min**	14(26.92%)	8(28.57%)	11 (61.11%)	3(50%)
**Total**	52(100%)	28(100%)	18(100%)	6(100%)
**Mean ± SD**	**2.67 ± 0.86**	**2.57 ± 0.68**	**5.37 ± 0.72**	**2.75 ± 1.06**
**chi square**	**8.02**	**df=3**	**p-value=0.05(NS)**	

However, the mean clotting time of the gestational hypertension patients was 6.6 min, in patients with mild preeclampsia was 6.3 min and severe preeclampsia patients was 6.2 min. The clotting time was more than 5 min in 35.7% cases of gestational hypertension and was found to be 66.67% of the mild preeclampsia patients and severe preeclampsia patients. This difference in clotting time was not found to be statistically significant between the patients with gestational hypertension, mild preeclampsia and severe pre-eclampsia (Table 8).

**Table 8:** Shows distribution of the study groups along with control group according to Clotting time.

Clotting time	Normal Pregnancy n (%)	Gestational hypertension n (%)	Mild Preeclampsia n (%)	Severe Preeclampsia n (%)
**< 5 min**	34(65.38%)	18(64.29%)	6(33.33%)	2(33.33%)
**>5 min**	18(34.62%)	10(35.71%)	12 (66.67%)	4(66.67%)
**Total**	52(100%)	28(100%)	18(100%)	6(100%)
**Mean ± SD**	5.25 ± 0.68	6.69 ± 0.78	6.39 ± 0.53	6.25 ± 0.35
**Chi square**	**7.59**	**df=3**	**p-value=0.06(NS)**	

## 4 Discussion

Hypertensive disorders are one of the most important causes of perinatal and maternal mortality and morbidity worldwide. A variety of hematological changes are observed in them with thrombocytopenia being the most common one. Moreover, derangements in coagulation and fibrinolytic system can occur in pregnancy causing a hypercoagulable state. In these patients, to rule out DIC and HELLP syndrome, a coagulation profile needs to be done.

In our study, maximum number of cases of mild preeclampsia were seen in the age group of 26–30 years (55%) followed by 33% cases in 20–25 years. Among severe preeclampsia category, maximum cases (66%) were between 20 and 25 years. Our age related data correlated well with study conducted by Chaware et al. [7]. The highest prevalence of gestational hypertension was found to be in primigravida (about 23%).

The mean platelet count in our study in control group, at term pregnancy was found to be 2.85 ± 0.73 lakhs/cumm whereas the mean platelets in gestational hypertension study group was 2.01 ± 0.54 lakhs/cumm and was 1.81 ± 0.46 lakhs/cumm in mild preeclampsia and 1.66 ± 0.85 lakhs/cumm in severe eclampsia cases, indicating a decrease in mean platelet counts with the increasing grades of PIH. A significant decrease in platelet count (p < 0.0001) was noted between the gestational hypertension, mild preeclampsia and severe preeclampsia study groups. Decreasing platelet count values observed in our study with increasing grades of PIH was consistent with the studies conducted by S. Mohapatra et al., Shete Anjali et al., Bhia Namavar Jahromi et al., Sameer et al., Ellora devi et al., Priyanka Chauhan et al. and [2,[8], [9], [10], [11], [12]].

A very few studies have been conducted regarding the probability of using MPV as an adverse outcome indicator in PIH cases and have shown variable results. Some studies have shown macro thrombocytosis and increased mean platelet volume in patients with moderate or severe hypertension in pregnancy [13], whereas some studies have shown that there was no change in the mean platelet volume in patients with mild to moderate hypertension [14]. Mean MPV in the normotensive pregnant females in our study at term was observed to be 8.47 ± 0.93 fl (Range of 8–12) and showed an increase in mean values with increasing grades in mean values with mean of 8.65 ± 0.97 in gestational hypertension cases and 8.79 ± 0.68 in mild preeclampsia and 10.33 ± 1.07 in severe preeclampsia cases indicating an increasing but not significant (p = 0.07) trend of increased values with increasing grades of pregnancy induced hypertension cases. Change in MPV happened with the increasing grades of pregnancy. In present study we found mild increase in MPV values from normotensive pregnant women to eclampsia patients which correlated with other studies.

Various studies validate PDW as a good marker of platelet dysfunction in PIH cases with variable results. In our study the mean PDW in normal cases was 10.90 ± 1.32 fl and showed an increase with progression of disease in PIH cases with value of 11.33 ± 1.11 fl in gestational hypertension cases and 11.96 ± 0.77 fl in mild preeclampsia and with mean of 11.7 ± 0.14 fl in severe eclampsia cases. Although the mean PDW values increased with the increasing grades of PIH but it was not found to be statistically significant. Bhavna Thakur et al. also studied PDW in PIH cases and observed the mean of 13.7 fl in Gestational hypertension cases and stated mean of 15.66 fl preeclampsia cases [15]. In gestational hypertension cases PDW was found to be non-significant but was found to be significant in pre-eclampsia group. Non-significant difference in PDW in the present study correlated well with Doğan et al. study which also found non-significant difference in PDW among women in study groups of severe preeclampsia, mild preeclampsia, and healthy controls [16]. A generalized observation was that mean PDW showed increased values as compared to the control group with increasing severity of PIH indicating that increased but not significant values of MPV and PDW can give an edge in signifying the various grades of PIH.

In present study the mean prothrombin time of patients with gestational hypertension was 16.59 ± 1.44 s, in mild preeclampsia it was 17.61 ± 2.88 s and in severe eclampsia it was found to be 18.88 ± 0.0 s. 57.14% of gestational hypertension, 83.33% of mild preeclampsia cases and all cases of severe eclampsia had prolonged prothrombin time with significant P-value <0.0001. Our study data correlated well with the following statistically significant studies for PT. Study by Priyadarshini et al. observed the mean prothrombin time of 15.27 s in patients with preeclampsia and found it to be 13.72 s in cases with normal pregnancy [17]. The increase in PT in severe eclampsia cases was found to be statistically significant. Similarly study by Tashin Mushtaque et al. study revealed the mean prothrombin time in three study groups for normal pregnancy, non-severe PIH and severe PIH patients were 10.9 s, 10.1 s and 9.8 s respectively, with significant P-value less than 0.0001 [18]. In a study conducted by Mishra et al. it was observed that the mean PT was found to be significantly increased with p value of <0.05 in severe preeclampsia and eclampsia study groups [19]. Shetty et al. studied haematological changes in pregnancy-induced hypertension and observed that the 28.57% cases of mild PIH and 82.86% of severe PIH had prolonged PT (>14 s). The PT in severe PIH was found to be significantly prolonged (P < 0.05) [20]. Abdulla et al. studied PT in 100 PIH cases and found that 50% of cases have prolonged PT with significant p value of (0.000) indicating that a highly significant difference in prothrombin time occurs between cases and their control [5].

In present study the mean APTT for normal pregnancy, gestational hypertension and mild preeclampsia and severe eclampsia were 25.76 ± 2.99 s, 33.55 ± 2.44 s,38.79 ± 2.52 s and 41.85 s respectively, with significant P-value <0.0001. Study done by Tashin Mushtaque et al. revealed the mean APTT for normal pregnancy, Non severe PIH and severe PIH patients were 26.68 s, seconds 26.71 and 26.25 s respectively, with P value less than 0.005 was statistically significant [18].

The Bleeding time was found to be prolonged for more than 5 min in 28.57% of the gestational hypertension study group, 61.11% of the mild preeclampsia group and was found to be increased in 50% of the patients of severe eclampsia. In gestational hypertension study groups the majority of cases 71.43% showed normal bleeding time of below 5 min but in mild preeclampsia cases the majority of 61.11% of cases showed an increase towards the upper limit of the normal with 50% of the cases showed an increase in BT in severe preeclampsia cases, although the BT levels showed an increase in levels towards increasing grade of hypertension but the results were found to be statistically insignificant. Our study results were similar to study by chaware et al. [7] and Chavan et al. [21].

In our study the mean clotting time of the gestational hypertension patients was 6.69 min, in patients with mild preeclampsia was 6.39 min and in severe preeclampsia patients it was 6.25 min. The clotting time was more than 5 min in 35.71% gestational hypertension patients, 66.67% of the mild and severe preeclampsia patients. In gestational hypertension study groups the majority of cases 64.29% showed normal bleeding time of below 5 min but in both mild and severe preeclampsia cases study groups the majority of 66.67% of cases showed an increase towards the upper limit of the normal, although the CT levels showed an increase in levels towards increasing grade of hypertension but the results were found to be statistically insignificant. Our study results were similar to study by chaware et al. [7] and Anjali et al. [8].

During data analysis, the reference range showed variations between different studies suggesting that every laboratory should establish their own reference intervals and cut off values. Most of the studies indicated platelet counts as an important predictor in assessing the severity of PIH cases. Significant prolongation of PT, APTT and significant fall in platelet counts was observed in PIH study groups indicating derangements in coagulation cascade in PIH.

## 5 Conclusion

In the present study, platelet counts had inverse relationship and PT; APTT had direct relationship with increasing severity of pregnancy induced hypertension. The coagulation abnormalities like HELLP syndrome and DIC are major causes of maternal deaths amongst PIH cases. Data observed from present study can be helpful in identifying the abnormalities in platelet parameters and coagulation profile in relation to PIH cases at an earlier stage and can prove to be helpful in management of complications arising in relation to PIH and thus can help in reduction of maternal and foetal mortality.

## Ethical approval

Approved by IEC VIA LETTER NO.

DEAN/NORTH DMC/MC/2021/1031 DATED:27/12/2021.

## Sources of funding

NIL.

## Author contribution

1. Dr. Namita Bhutani : HAVE WRITTEN THE MANUSCRIPT. 2. DR. Varuna Jethani: ASSISTED IN EDITING OF THE MANUSCRIPT. 3. Dr. Sumit Jethani: SUPERVISION OF THE STUDY. 4. Dr. Karuna Ratwani: DATA COLLECTION AND SUPERVISION OF THE STUDY.

## Registration of research studies

Approved by IEC VIA LETTER NO. DEAN/NORTH DMC/MC/2021/1031 DATED:27/12/2021.

## Guarantor

DR. Namita Bhutani.

## Consent

Written informed consent was obtained from the patients for publication of this study and accompanying images. a copy of the written consent is available for review by the editor–in–chief of this journal on request.

## Data availability statement

Data will be available to all the readers as per journal's rules.

## Provenance and peer review

Not commissioned, externally peer-reviewed.

## Declaration of competing interest

NIL.
